# *Clostridium difficile* Infections in Medical Intensive Care Units of a Medical Center in Southern Taiwan: Variable Seasonality and Disease Severity

**DOI:** 10.1371/journal.pone.0160760

**Published:** 2016-08-10

**Authors:** Jen-Chieh Lee, Yuan-Pin Hung, Hsiao-Ju Lin, Pei-Jane Tsai, Wen-Chien Ko

**Affiliations:** 1 Department of Internal Medicine, National Cheng Kung University Hospital, College of Medicine, National Cheng Kung University, Tainan, Taiwan; 2 Department of Internal Medicine, Ministry of Health and Welfare, Tainan, Taiwan; 3 Graduate Institute of Clinical Medicine, National Health Research Institutes, Tainan, Taiwan; 4 Department of Medical Laboratory Science and Biotechnology, College of Medicine, National Cheng Kung University, Tainan, Taiwan; 5 Center of Infection Control, National Cheng Kung University Hospital, Tainan, Taiwan; Cleveland Clinic, UNITED STATES

## Abstract

Critical patients are susceptible to *Clostridium difficile* infections (CDIs), which cause significant morbidity and mortality in the hospital. In Taiwan, the epidemiology of CDI in intensive care units (ICUs) is not well understood. This study was aimed to describe the incidence and the characteristics of CDI in the ICUs of a medical center in southern Taiwan. Adult patients with diarrhea but without colostomy/colectomy or laxative use were enrolled. Stool samples were collected with or without 5 ml alcohol and were plated on cycloserine-cefoxitin-fructose agar. *C*. *difficile* identification was confirmed by polymerase chain reaction. There were 1,551 patients admitted to ICUs, 1,488 screened, and 145 with diarrhea. A total of 75 patients were excluded due either to laxative use, a lack of stool samples, or refusal. Overall, 70 patients were included, and 14 (20%) were diagnosed with CDI, with an incidence of 8.8 cases per 10,000 patient-days. The incidence of CDI was found to be highest in March 2013 and lowest in the last quarter of 2013. The cases were categorized as the following: 5 severe, complicated, 5 severe, and 4 mild or moderate diseases. Among the 14 cases of CDI, the median patient age was 74 (range: 47–94) years, and the median time from admission to diarrhea onset was 16.5 (4–53) days. Eight cases received antimicrobial treatment (primarily metronidazole), and the time to diarrheal resolution was 11.5 days. Though 6 cases were left untreated, no patients died of CDI. The in-hospital mortality of CDI cases was 50%, similar to that of patients without CDI (46.4%; *P* = 1.0). We concluded that the overall incidence of CDI in our medical ICUs was low and there were variable seasonal incidences and disease severities of CDI.

## Introduction

Diarrhea is common in critically ill patients, with a reported prevalence rate of 14–21% [1]. It is a challenge for physicians in intensive care units (ICUs) due to its variety of etiologies and its complex relationship with underlying illnesses. The etiology of diarrhea can be classified as infectious or non-infectious [1]. *Clostridium difficile* infection (CDI) is the leading cause of infectious diarrhea in hospitals [2].

*C*. *difficile* is a gram-positive, spore-forming, anaerobic gut pathogen [3]. Many details of the complex pathogenesis of CDI remain under investigation, such as the receptors that mediate toxin endocytosis, the pathogenic role of binary toxin, the innate immune responses that affect the course of CDI, and the factors related to the sporulation and germination of *C*. *difficile* in the intestine [3]. Common risk factors of CDI include increased illness severity, increased age, antibiotic exposure, and gastric acid suppression [4]. Immunocompromised comorbidities (*i*.*e*., neoplastic diseases, antineoplastic chemotherapies, diabetes mellitus, or organ transplantation) [4–6], hypoalbuminemia [4,5], prolonged hospital stay [5], mechanical ventilation [4], and tube feeding [7] may predispose patients to CDI. Critically ill patients often share one or some of the above factors, and thus the incidence of CDI in ICUs is expected to be high.

The reported incidence of CDI in the ICUs of North America and Europe was 8.7–53.9 cases per 10,000 patient-days [8–12], or 0.4–4% of the ICU population [12–17]. A recent meta-analysis revealed a global CDI incidence rate of 2% among critically ill patients [18]. Epidemiological data of CDI from Asian nations, including Taiwan, are limited [18, 19]. Moreover, seasonal variations in CDI have been reported in Australia, Canada, and the United States [20–24]. An association between the fluctuation in CDI incidence and viral respiratory tract infections during the winter months has been proposed in several studies [21, 23, 24]. More antibiotics are prescribed for presumed bacterial infections of the lower respiratory tract during this period. The time lag from antibiotic exposure to CDI development was estimated to be one to two months [25]. The peak CDI incidence is expected to occur during the spring/summer months. The degree of seasonality of CDI incidences in Asian countries is unclear. The present study aims to investigate seasonal variations in CDI incidence and its clinical manifestations, including disease severity, in ICU patients.

## Materials and Methods

A prospective study conducted in three medical ICUs (a total of 42 beds) of National Cheng Kung University Hospital (NCKUH), a medical center in southern Taiwan, was approved by the Institutional Review Broad (IRB) of the study hospital (B-ER-101-284). All patients (or their legal representatives) gave their written informed consent. From March 1, 2013 to March 31, 2014, the stool output data of ICU patients in the clinical information system (ICIP, e-Edition, PHILIPS) were screened daily. Inclusion criteria were as follows: 1) adult patients at least 20 years of age, 2) no prior colostomy/colectomy, and 3) the presentation of diarrhea at least 72 hours after ICU admission that persisted for at least 48 hours. Diarrhea was defined as at least three recorded occurrences of loose or watery stool per day, and the resolution of diarrhea was defined as less than three passages of loose or watery stool per day for at least 48 hours. Patients using laxatives concurrently or within two days before diarrhea onset were excluded. Stool samples were plated on cycloserine-cefoxitin- fructose agar (CCFA, Creative Life Science, LTD, Taiwan) and cultured under anaerobic conditions. Isolates were identified as *C*. *difficile* by odor, colony morphology, and biochemical reactions; the presence of *tcdB* in *C*. *difficile* isolates was assessed by polymerase chain reaction [26]. CDI was defined as the presence of diarrhea and *tcdB*-carrying *C*. *difficile* in the stools in the absence of other diarrhea causes.

Electronic medical charts were reviewed to obtain demographic data, clinical signs and symptoms, current or recent medications, the duration of hospital or ICU stays, and laboratory findings. The incidence rate of CDI is presented as CDI cases per 10,000 patient-days of ICU stays. CDI incidence rates during the study period and each quarter were calculated. The severity of CDI was stratified as follows: 1) mild or moderate defined by a white blood cell (WBC) count ≤15,000 cells/μL and a serum creatinine change <1.5 times the premorbid level, 2) severe defined by a WBC count >15,000 cells/μL or a serum creatinine change ≥ 1.5 times the premorbid level, and 3) severe, complicated defined by the occurrence of hypotension/shock, ileus, or megacolon, as suggested by the Society for Healthcare Epidemiology of America and Infectious Disease Society of America (SHEA/IDSA) [27].

Recent hospitalization was defined as prior admission to a hospital within three months before CDI onset. The healthcare facilities from which the patients were transferred were either nursing homes or respiratory care wards. Previous antibiotic exposure was traced in the electronic medical system or transference records and defined as antibiotics prescribed within one month before diarrhea onset. Concurrent fecal colonization of vancomycin-resistant enterococci (VRE) was defined as the presence of VRE isolates in stools also positive for toxigenic *C*. *difficile* isolates from patients without proven VRE infections. Systemic inflammatory response syndrome (SIRS) [28], Acute Physiological and Chronic Health Evaluation II (APACHE II) score [29], and Charlson’s index [30] were defined as previously described. *C*. *difficile*-specific treatment refers to parenteral or oral metronidazole or oral vancomycin alone or in combination.

Additionally, CDI incidence data in NCKUH and Tainan Hospital (TH), a regional hospital two kilometers from NCKUH, were obtained from the clinical microbiology laboratory during the study period. Enzyme immunoassays (EIA) for *C*. *difficile* toxin (NCKUH: Premier^®^ Toxins A&B, Meridian Bioscience, Inc., Cincinnati; TH: C. Diff Quik Chek Complete^®^, Alere North America, Inc., Orlando) were performed in suspected CDI cases according to the discretion of the attending physicians, and thus IRB approval for such incidence information was waived.

All data were processed by the SPSS 20 edition. Continuous data were expressed as the means ± standard deviations. The χ^2^ test or Fisher’s test was used for categorical variables and Student’s t-test was used for continuous variables. A two-tailed *P* value of less than 0.05 was considered to be statistically significant.

## Results

**Fig 1:**
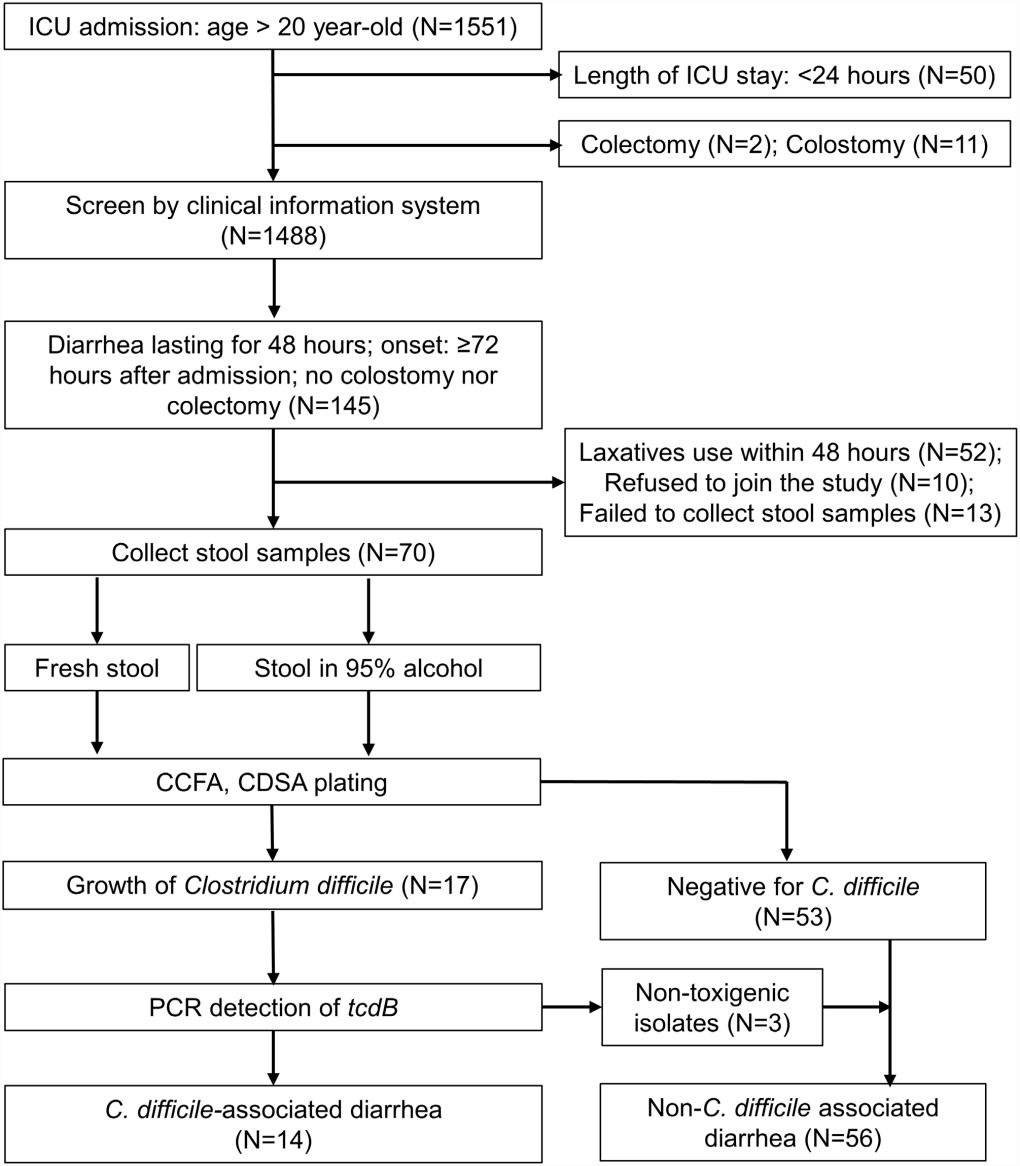
Patient flowchart of *Clostridium difficile* infection in ICUs. CCFA = cycloserine-cefoxitin fructose agar; CDSA *= Clostridium difficile* selective agar; PCR = polymerase chain reaction.

Throughout the study period, 1,551 patients were admitted to three ICUs. With the exclusion of 50 patients that died within the first 24 hours after admission, 11 patients with colostomies, and two patients with colectomies, a total of 1,488 patients were screened for enrollment. A total of 145 patients developed diarrhea, but 52 were excluded due to laxative use. Among the 93 study candidates, 10 refused to join the study, and stool samples of 13 patients were not available. Finally, 70 (75.3%) diarrhea patients were enrolled for stool cultures. *C*. *difficile* was isolated from 17 (24.3%) samples, among which 14 (20%) isolates were tested positive for *tcdB*, the putative cause of CDI (Fig 1).

**Fig 2:**
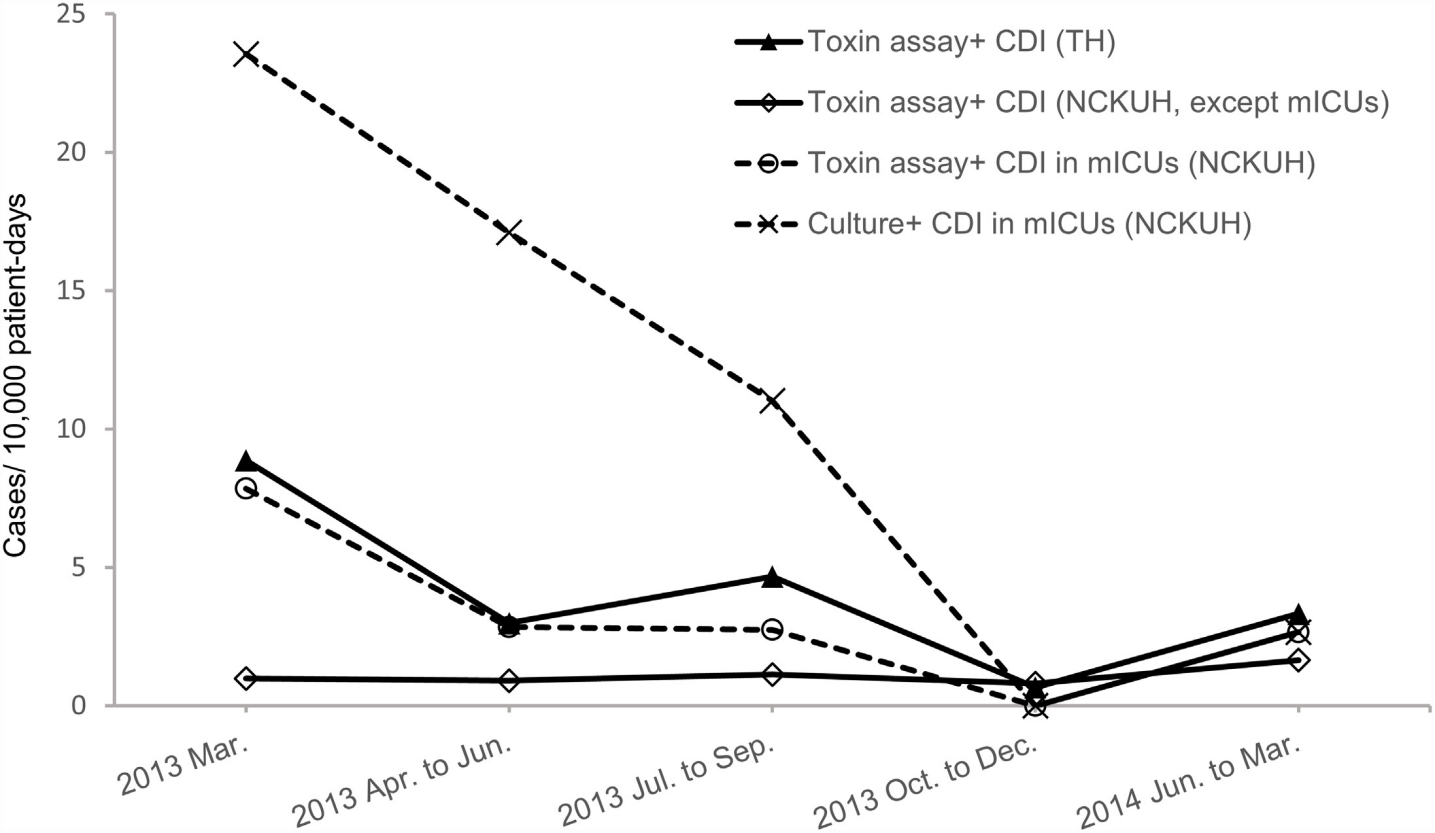
The incidences of *Clostridium difficile* infection (CDI) in different clinical settings. The incidences of CDI in a medical center (NCKUH) and a regional hospital (TH) between March 2013 and March 2014. CDI was diagnosed by the presence of toxigenic *C*. *difficile* isolates (Culture+) or *C*. *difficile* toxin (Toxin+) in stools. mICUs = medical intensive care units.

The overall incidence of CDI diagnosed by stool culture was 8.8 cases per 10,000 patient-days in medical ICUs. The incidence rate of CDI was highest in the first month of the study (March of 2013) at 23.6 cases per 10,000 patient-days, and the incidence rate was lowest during the last quarter of 2013, wherein no cases were discovered (Fig 2). Likewise, the incidence rate of CDI in the NCKUH (excluding medical ICUs) was low, at 1.1 cases per 10,000 patient-days, and remained stable throughout the study period (Fig 2). Similar data from Tainan Hospital, where CDI cases were diagnosed by toxin EIA, showed a similar pattern, with the highest incidence rate observed in March of 2013 (8.9 cases per 10,000 patient-days) and the lowest rate observed in the last quarter of 2013 (0.7 cases per 10,000 patient-days). Overall, we observed a seasonal variation of CDI incidence in two hospitals, irrespective of the diagnostic assay used.

Demographic information, the length of hospital/ICU stay, the types and severities of any comorbidities, antibiotic exposure, VRE colonization, and in-hospital mortality in 14 patients with CDI were not different from those of 56 patients with diarrhea but with no confirmed CDI (Table 1). Among the 14 cases of CDI, the median age was 74 years (range: 47–94 years), and nine were males. The median time from hospital admission to diarrhea onset was 16.5 days (range: 4–53 days). The median APACHE II score was 17 at enrollment.

**Table 1:** Characteristics of the patients with or without *Clostridium difficile* infection (CDI) in intensive care units (ICUs).

Variables	Case number (%)		*P* values
	CDI (n = 14)	Non-CDI (n = 56)	
Age, median (IQR)	75 (63–79)	71 (56.3–83)	0.414
Male gender	9 (64.3)	29 (51.8)	0.589
Recent hospitalization	9 (64.3)	34 (60.7)	1
Referral from healthcare facilities	2 (14.3)	14 (25.0)	0.497
BMI admission, mean ± standard deviation (SD)	23.7 ± 4.3	21.6 ± 5.0	0.066
BMI inclusion, mean ± SD	23.9 ± 4.2	22.3 ± 4.8	0.23
Days from hospital admission to diarrhea onset, median (IQR)	16.5 (10.8–29.8)	12.5 (8–21.8)	0.243
Days from ICU admission to diarrhea onset, median (IQR)	10.5 (4.5–17.3)	9 (5–13.8)	0.803
Days of total ICU stay, median (IQR)	22 (13.5–28.8)	25.5 (12.3–35.8)	0.654
Comorbidities			
Chronic obstructive pulmonary disease	0 (0)	7 (12.5)	0.331
Liver cirrhosis	2 (14.3)	8 (14.3)	1
End-stage renal disease	3 (21.4)	5 (8.9)	0.193
Chronic heart disease	3 (21.4)	11 (19.6)	1
Bedridden	4 (28.6)	19 (33.9)	1
Diabetes mellitus	6 (42.9)	23 (41.1)	1
Cancer	6 (42.9)	16 (28.6)	0.344
Charlson's index, median (IQR)	6 (3.3)	7 (4.0)	0.667
Antibiotic exposure within one month before diarrhea onset	14 (100)	56 (100)	1
Glycopeptide	9 (64.3)	23 (41.1)	0.208
Penicillin	9 (64.3)	38 (67.9)	1
Third generation cephalosporin	9 (64.3)	28 (50.0)	0.51
Fourth generation cephalosporin	2 (14.3)	18 (32.1)	0.321
Carbapenem	6 (42.9)	20 (35.7)	0.853
Fluoroquinolone	5 (35.7)	22 (39.3)	1
Antifungal therapy	3 (21.4)	17 (30.4)	0.742
Symptoms and signs at enrollment			
Systemic inflammatory response syndrome	14 (100)	51 (91.1)	0.575
Body temperature >38°C	8 (57.1)	29 (51.8)	0.952
Body temperature <36°C	11 (78.6)	29 (51.8)	0.131
Abdominal distension	8 (57.1)	22 (39.3)	0.365
APACHE II score			
At admission, median (IQR)	24 (19–29.3)	24 (16.3–26.8)	0.431
At enrollment, median (IQR)	17 (13–26)	19 (14.3–25)	0.613
Concomitant VRE colonization	2 (14.3)	24 (42.9)	0.095
In-hospital mortality	7 (50.0)	26 (46.4)	1

IQR: interquartile range, 25th percentile—75th percentile; BMI: body mass index; APACHE: Acute physiological and chronic health evaluation; VRE: vancomycin-resistant enterococci.

As stratified by the SHEA/IDSA guidelines [27], there were 5 severe, complicated cases, 5 severe cases, and 4 mild or moderate cases of CDI (Table 2). Eight patients received antimicrobial treatment for CDI, and four severe cases were treated by oral metronidazole. All treated patients recovered from diarrhea with a median time of 11.5 days after antimicrobial intervention. Six patients were not treated. A case of severe CDI and two cases of severe, complicated CDI were self-limited without antibiotic therapy. Seven CDI patients died during hospitalization, but the cause of death was not CDI for any these patients.

**Table 2:** Clinical characteristics of 14 cases of *Clostridium difficile* infection (CDI).

Case No.	SHEA/IDSA, CDI severity	APACHE II (enrollment)	Diarrhea onset to CDI treatment (day)	CDI therapies (days)			Diarrhea duration (day)	Diarrhea onset to in-hospital death (day)
				Regimen 1	Regimen 2	Regimen 3		
1	Severe, complicated	29	5	IV metronidazole (5)	Oral vancomycin (17)	Oral vancomycin + IV metronidazole (10)	33	100
2	Severe, complicated	26	11	IV metronidazole (6)	Oral metronidazole (10)		33	-
3	Severe	11	1	Oral metronidazole (10)			10	64
4	Severe	17	2	Oral metronidazole (10)			8	58
5	Severe	14	6	Oral metronidazole (10)			10	-
6	Severe	13	4	Oral metronidazole (10)			7	-
7	Mild or moderate	10	10	Oral metronidazole (7)			26	28
8	Mild or moderate	13	9	Oral metronidazole (10)			23	-
9	Severe, complicated	26	-	No *C*. *difficile*-specific therapy			4	4
10	Severe, complicated	33	-	No *C*. *difficile*-specific therapy			7	52
11	Severe, complicated	19	-	No *C*. *difficile*-specific therapy			11	-
12	Severe	17	-	No *C*. *difficile*-specific therapy			10	-
13	Mild or moderate	21	-	No *C*. *difficile*-specific therapy			7	14
14	Mild or moderate	14	-	No *C*. *difficile*-specific therapy			14	-

*APACHE = Acute Physiology and Chronic Health Evaluation; SHEA/IDSA = Society for Healthcare Epidemiology of America/Infection Disease Society of America; IV = intravenous.

## Discussion

The reported incidence rate of CDI in medical and/or surgical ICUs, ranging from 8.7 to 53.9 cases per 10,000 patient-days or 1.5–4.8% of ICU population [8–10, 13–15, 19], was found to be higher than those in various subgroups, such as neurosurgical, burn, or cardiothoracic ICUs (from 7.9 to 8.3 cases per 10,000 patient-days or 0.5 to 0.6% of ICU population) [11, 12, 16, 17]. Such variability may be related to differences in patient populations, underlying illness severities, comorbidities, or antibiotic use among different ICUs. The incidence of CDI in our medical ICUs (8.8 cases per 10,000 patient-days or 0.9% ICU admissions) was close to the lower end of the incidence range observed in general ICUs, but lower than the global pooled rate of 2% found from 22 published articles encompassing 80,835 ICU patients [18]. There were three articles reporting CDI incidence rates in Taiwan. Due to variable study designs, diagnostic methods, and target populations, the CDI incidence rate ranged from 4.3 to 10.1 cases per 10,000 patient-days [31–33]. Among these publications, one retrospective study specifically indicated a high CDI incidence in ICUs at 11.1 cases per 10,000 patient-days [32]. Though these figures do not completely represent the general patient population, they indicate that CDI is not uncommon in Taiwanese hospitals.

Our study shows that the incidence of CDI from March through June was higher than that from October to December. These months are equivalent to spring and late autumn in Taiwan, respectively. Such an incidence change was also present in another hospital in Tainan City. A recent systemic review summarized the patterns of seasonal variation in both the Northern and Southern Hemispheres [34]. However, CDI incidence data from Asia are scarce in the literature. The seasonal pattern in southern Taiwan was found to be similar to that observed in the Northern Hemisphere, peaking in the first quarter. The incidence of CDI in the Taiwan Island warrants further investigation.

In this study, we failed to recognize any clinical features of CDI that were significantly different from those of other causes of diarrhea in the ICU. Though they are known risk factors of CDI, neither preexisting comorbidities nor antibiotic exposure were found to be distinctive. The sample size in our study is likely too small to detect distinguished clinical variables. However, ICU admission *per se* may inadvertently cause a bias towards patients with debilitating comorbidities and/or multiple antibiotic exposures. Thus, the subjects were somewhat homogeneous, and potential differences may have been obscured. Nevertheless, there was a potential distinction in host characteristics among those with or without CDI. Cancer and end-stage renal disease, which is more prevalent in CDI patients, are traditional risk factors of CDI, due to weakened immune responses and certain medications (either acid suppression therapy, antibiotics, or chemotherapy) [35, 36]. Not unexpectedly, recent exposure to glycopeptides [4, 37], broad-spectrum cephalosporins [38], or carbapenems [38] has a notorious effect in predisposing patients to CDI development. CDI has been associated with VRE colonization; Fujitani *et al*. reported VRE colonization in 56% of 158 patients with CDI [39]. However, our data showed a contradictory result wherein the VRE colonization rate was found to be lower in CDI cases than non-CDI cases (14.3% *vs*. 42.9%). The interaction between fecal VRE colonization and *C*. *difficile* or CDI warrants further investigation, as both signify prior heavy antibiotic exposure.

Among the three patients with severe, complicated CDI that did not receive *C*. *difficile*-specific therapy, one died before the stool culture result became available, and two experienced spontaneous resolution of their diarrhea illnesses. Moreover, four cases of severe CDI were successfully managed by oral metronidazole, and a single case was managed by no specific therapy. These findings prompted the hypotheses that such “severe” or “severe, complicated” cases may in fact be mild cases of CDI complicated by other diseases that give rise to leukocytosis, azotemia, hypotension or shock. The resolution of severe or complicated CDI without guideline-recommended therapy also highlights the challenge in applying clinical severity scores to ICU patients with CDI. However, the likelihood of severe complications from CDI in diarrhea patients should always be a concern, and the proper implementation of CDI treatment guidelines should be emphasized [40].

This study comes with several limitations. The data were collected from a single center over a short period of time with limited case numbers. The timing of laboratory screening for CDI is difficult to optimize in clinical prospective studies, but here we actively surveyed CDI cases with the aid of an electronic medical system, thereby minimizing the loss of potential study candidates. Furthermore, we only included cases with new-onset diarrheal illnesses, and those with other relatively rare manifestations of CDI, such as ileus or sepsis, were not considered in our work. However, published clinical studies of CDI often include diarrhea as the primary symptom of CDI [8, 10, 12–14, 17, 19]. Additionally, we only had access to the prescription data in our hospital, transference records, or patient self-reports, and drug information in other clinics or hospitals was incomplete. This limitation may bias the impact of prior medication on CDI development in ICU patients.

In conclusion, the incidence of CDI in our medical ICUs was found to be in the low end of the range determined from published data obtained in other regions, and there was seasonal variation in CDI incidence and differing disease severities. Further investigations with extended study durations and populations are warranted to explore the clinical impact of CDI among critically ill patients in ICUs.

## Supporting Information

S1 File The dataset of 70 enrolled cases. (XLSX)

S1 Table Toxin genotyping characteristics of 14 toxigenic *Clostridium difficile* strains. (DOCX)
