# Supplementary material for: Clostridium difficile Infections in Medical Intensive Care Units of a Medical Center in Southern Taiwan: Variable Seasonality and Disease Severity
Source: PLoS One. 2016 Aug 10;11(8):e0160760. doi: 10.1371/journal.pone.0160760 (PMC4979958; doi:10.1371/journal.pone.0160760)
Supplement: S1 Table — (DOCX) [file pone.0160760.s002.docx]

**S1 Table.** Toxin genotyping characteristics of 14 toxigenic *Clostridium difficile* strains.

| Case No.* | *tcdA* | *tcdB* | *cdtA* | *cdtB* | 16S | *tcdC* |
| --- | --- | --- | --- | --- | --- | --- |
| 1 | + | + | - | - | + | Wild type |
| 2 | + | + | - | - | + | Wild type |
| 3 | + | + | - | - | + | Wild type |
| 4 | + | + | - | - | + | Wild type |
| 5 | + | + | - | - | + | Wild type |
| 6 | + | + | - | - | + | Wild type |
| 7 | + | + | - | - | + | Wild type |
| 8 | + | + | - | - | + | Wild type |
| 9 | + | + | - | - | + | Wild type |
| 10 | + | + | + | + | + | Deletion |
| 11 | + | + | - | - | + | Wild type |
| 12 | + | + | - | - | + | Wild type |
| 13 | + | + | - | - | + | Wild type |
| 14 | + | + | - | - | + | Wild type |

*Causative isolate in the corresponding case number in Table 2.
